# A Comprehensive Search for Recombinogenic Motifs in the Human Genome

**DOI:** 10.1371/journal.pone.0062920

**Published:** 2013-04-23

**Authors:** Henry R. Johnston, David J. Cutler

**Affiliations:** 1 McKusick-Nathans Institute of Genetic Medicine, Johns Hopkins University School of Medicine, Baltimore, Maryland, United States of America; 2 Department of Human Genetics, Emory University School of Medicine, Atlanta, Georgia, United States of America; Emory University School of Medicine, United States of America

## Abstract

The patterns of male and female recombination vary greatly on a macro scale. A unique motif in each gender, triggering a double strand break at its location, much in the way Chi sites operate in *E. coli*, could logically explain this difference. As such, we have undertaken a comprehensive search of all small motifs in an attempt to identify one or more that match to the available data. In the end, we conclude that no such motifs appear to exist in the human genome.

## Introduction

It has long been understood that in *Escherichia coli* the DNA sequence 5′-GCTGGTGG-3′, the so-called “Chi site,” is specifically recognized by the RecBCD complex, and this short sequence is instrumental to localizing homologous recombination in bacteria [1]. Encountering the Chi site appears to convert RecBCD's behavior from DNA destruction to facilitating homologous recombination, and as a result the position of Chi sites is intimately tied to the position of homologous recombination events in *E. Coli*. The presence of a Chi site is mechanistically required in an *E. Coli* region experiencing recombination, and regions of that genome experiencing recombination will necessarily contain a Chi site. Whether or not there is a motif present in the human genome that localizes to recombination sites remains an open question.

Previous work has identified a degenerate 13-mer motif [2] that is statistically enriched in regions undergoing recombination in humans. These authors show that approximately 50% of LD-defined hotspots contain this motif, far more than expected by chance. However, this motif is neither necessary nor sufficient for the generation of recombination events, as half of LD-defined recombination hotspots do not contain this motif, and the vast majority of these motifs (89%) occur in regions of the genome not identified as recombination hotspots [3]. PRDM9 has been shown to be a mediator of recombination in humans [4], [5], [6], and the degenerate 13-mer is a predicted binding motif for a specific *PRDM9* allele. In sperm typing studies, however, the 13-mer has not been shown to be a direct mediator of recombination [7]. Additionally, most recent work has demonstrated that the proposed PRDM9 binding sequence is not functional in our closest relative, chimpanzee [8].

The present work attempts to determine whether any motif has a similar distribution relative to recombination events in humans as the Chi site has in *E. Coli*. To do this, we ask whether any motif appears statistically associated with regions known to have experienced recombination events. We do this both locally, asking whether small genomic regions known to have undergone recombination are enriched for any motif, and globally asking whether the overall distribution of any motif matches the overall distribution of recombination rates across the human genome.

Three separate publically available datasets are used to address these questions. First, from an examination of the patterns of linkage disequilibrium in humans [9], regions of the genome have been identified that have experienced an unusually large numbers of recombination events in the history of humans [3]. Second, from examination of unusually large pedigrees [10] regions of the genome have been identified that experienced recombination in last few generations. Finally, from the patterns of segregation within pedigrees, a broad scale map of recombination rates across the genome is available [11].

There are two principal complications to this analysis. First, in order to detect a region experiencing a recombination event, DNA markers must be available that distinguish available alleles. The level of variation in humans is such [12] that resolution of recombination events is never better than kilobase scales [9] and for recent events often no better than 100 s of kb [10] scales, and for broad patterns megabase scales [13]. Second, the broad scale of recombination shows vast differences between males and females [11], [13]. There is far more recombination in females than males, and the patterns of those events differ. Female recombination appears fundamentally uniformly distributed along chromosome arms, whereas male recombination is usually concentrated towards the telomeric end of chromosomes. This suggests the possibility any motif associated with recombination might be sex specific.

To do these statistical analyses we examine all possible short motifs, as well as all possible combinations of two short motifs in close proximity to one another. While several sequences show promise, and an enormous statistical enrichment in one or more data sets, we are unable to find any motif that can explain both local and global patterns of recombination in the genome. We believe that humans may simply lack any motif whose function is as intimately tied to recombination as Chi is in *E. Coli*.

## Methods

The most basic approach to identifying a recombination causing motif is an analysis of regions known to have experienced more recombination events than surrounding regions. The key limitation to inferring the position of recombination events involves informative markers [3]. A recombination event occurs at some position. In order to detect that event, an “informative” marker, in this case [10] a SNP that is heterozygous in relevant samples, must exist on either side of the event. Thus, the precise event is not detectable, but instead a “window” that contains the event is found. That window includes the precise position of the recombination event, and extends 5′ and 3′ away from that event until an informative marker is detected. One expects the position of the actual recombination event to be on average near the center of windows, assuming the distance to the nearest 5′ informative marker is on average the same as the distance to the nearest 3′ informative marker. For each event individually, there is no known bias toward finding an informative marker more closely on the 5′ end or the 3′ end. Collectively, this both places the average recombination event in the center of the average recombination window as well as ensures that the distribution of all recombination events in all mapping windows has a significant level of clustering the centers of those windows. Thus, if a motif were to be highly specific and lead to the initiation of recombination, one might legitimately expect that it would be both overrepresented in regions enriched for recombination events, and the position of the motif ought to be disproportionately located towards the center of the window containing the exchange [3]. Two such data sets exist where local patterns of recombination can be analyzed. One, the set of linkage disequilibrium (LD)-defined hotspots identified by McVean et al, are known, at a minimum, to have had more recombination events in their histories than surrounding regions of the genome [3], [9]. The second, wherein individual recombination events have been mapped to recombination windows in a pedigree, can be further subdivided into those regions which contained a recombination event arising from female meiosis, and those arising from males [10].

To investigate whether a specific motif is generating these mapped recombination events, every possible 6, 7, 8 and 9-mer is tested to determine if any motif is enriched in these windows and if the position of the motif is clustered near the center of the window. A motif is said to be present, if either its sequence, or its reverse complement sequence matches the reference sequence at any position within a window. To determine center clustering, each window (LD-hotspot or mapped recombination event) is divided into 10 bins of equal size, and the number every possible 6, 7, 8, and 9-mer in each bin is counted. The sum of bins 5 and 6 (the center of a region) is then divided by the sum of bins 1 and 10 (the edges). Quotients larger than 1 for a given motif indicate clustering of that motif in the center of windows. Values less than 1 indicate an excess of motifs on the edges of windows. These values will be referred to as clustering scores from this point forward.

The experiment is then repeated in a set of matched random controls. For each window in the “real” data, 10 matched random windows are created. These matched random windows are generated to be the same size as the real window, and have GC content and SNP density [14] within 5% of the corresponding “real” window [3]. All possible 6, 7, 8 and 9-mers are counted in all random sets, with means and standard deviations calculated across the 10 replicated random windows. The motifs are sorted for overrepresentation in true windows. This is accomplished by dividing the total excess of motifs in real windows (the motif count in real windows minus the mean motif count in the random windows) by the standard deviation of the random windows. Thus the final statistic is analogous to a Z-score. Scores greater than 0 indicate an excess of the motif in real windows over random windows, and scores larger than 5 (five standard deviations) should generally be considered highly significant, even after multiple test correction for the vast numbers of motifs considered. This list of overrepresented motifs is further sorted by which motifs are found to be most clustered in the center of windows.

It should be noted here that the above data sets (hotspots and gender-specific recombination windows) are intentionally not combined in this analysis. Although on the surface that might seem to be a reasonable approach, when the relative sizes of the data sets are compared (Table 1), it is clear that hotspots have been identified on a very narrow scale, while the scale of recombination windows is massive. The noise from the large recombination windows would drown out any signal from hotspots. If we were to combine the 30,000+ hotspots with the few recombination windows of appropriate size, the large number of hotspots would overwhelm the analysis and the combined result wouldn't look significantly different from the hotspots alone. Consequently we have examined the data sets independently.

**Table 1:** Sizes of recombination mapping windows.

Widths	Hotspots	Male Windows	Female Windows
<5kb	22655	281	454
5–10kb	8030	319	465
10–30kb	2238	1344	1991
30–60kb	73	1536	2341
60–90kb	0	1141	1648
90–120kb	0	804	1270
>120kb	0	4166	6335
**Total Number**	32996	9591	14504
**Total Bases**	181943	3001938968	4973275771
**Average Width**	5.51 kb	312.9 kb	342.8 kb

This table shows the disparity in sizes between LD-identified hotspots and recombination mapping windows. Hotspots are finely localized, while mapping windows often span hundreds of kb.

On a global scale, it is expected that the distribution of a motif that causes recombination will match the global recombination rates and patterns seen in males and or females. As a result, all possible 6, 7, 8, and 9-mers are compared to the deCODE recombination maps of both genders [11]. The goodness of fit for the motif is calculated using the Kolmogorov-Smirnoff test statistic (K-S test) [15]. This statistic measures the maximum separation between two frequency distributions. P-values reported for this test indicate the likelihood that the two curves are drawn from the same distribution. Thus, for our analysis, high p-values indicate good concordance between the mapped curves while low p-values cause one to reject the null hypothesis that the curves are drawn from the same distribution. The K-S test statistics are added across all chromosome arms. With approximately 100 bins per chromosome arm, a K-S test statistic of 0.1 on an individual arm should generally be considered a very “good fit” between expected and observed, as it is not rejectable by chance at p = 0.1. Across 39 independent arms, a total K-S sum less than 3.9 should be considered a very good fit for all chromosomes. Values below 6 indicate generally good fits. Values above 10 should be considered atrocious.

A second potential method for identifying a recombination initiation motif on a global scale begins with the unique shape of the deCODE maps in males. Noting that chromosome arm 17q in males has the property of having 50% of its recombination occur in the telomeric 10% of the chromosome arm, it is posited that a male recombination motif ought to share this property. An analysis is conducted wherein every possible 6, 7, 8 and 9-mer is mapped to build 35 of the NCBI reference genome. Each motif has their K-S test score for chromosome 17q generated. The top matches are then condensed into a composite 6-mer. The motif is then built outward; adding any of the 4 bases on either side of the motif and determining which has the best K-S test score. This leads to an optimal motif. This motif is then compared to the remaining 38 male chromosome arm maps to complete the analysis on a global scale.

The same unique shape of male recombination maps that suggests the exploration of 17q also suggests the possibility that a pair of motifs spaced at some distance might be responsible for the initiation of recombination. The intuition here is that the shape of the recombination curves might be inducible by two different motifs, both nearly uniformly distributed, both sharing a slight excess near the telomeres, but which are together greatly enriched there. Thus, neither would look significant individually, but the pair, in fact, would. To this end, every possible combination of pairs of 6, 7 and 8-mers, at varying distances from 10 bp to 5 kb, are tested against the male deCODE map to determine which had the best fit, again using K-S test scores.

The previously identified 13-mer is neither necessary nor sufficient for the generation of a recombination event. Nevertheless, it might be uniquely positioned within hotspots and be the best available motif. As a result we tested the 13-mer in the exact same manners we tested all other 6, 7, 8, and 9-mers.

## Results

The number of tested motifs is enormously large. There are 2080, 8192, 32,897, and 131,072 unique 6, 7, 8 and 9-mers, respectively. When considering pairs of motifs, those numbers are approximately squared. Put most simply, no motif, or pair of motifs adequately explains the data. Many motifs are over-represented in the local analysis of recombination events. Many motifs are good fits for global patterns of female recombination. No motif is a good fit for global patterns of male recombination, and no motif looks uniquely clustered in the center of regions undergoing recent recombination in pedigrees. To demonstrate these broad conclusions we will present a small subset all of this analysis.

To illustrate the general result we focus on 6-mers. Because we consider a 6-mer present when either it, or its reverse complement is found on the forward-human strand, there are usually two choices for how to present any 6-mer. In all that follows we present the choice that is more G-rich (as opposed to C-rich), and when the two options have the same number of G's, we present the version that sorts first alphabetically.

Table 2 summarizes the results for the “best five” 6-mers in each of our separate data sets (LD-defined hotspots, female recombination windows, male recombination windows, global female map, and global male map). Focusing first on motifs enriched in LD-defined hotspots, we find all G rich 6-mers are dramatically enriched. GGGGGG itself is found at rates 68 standard deviations above random spots. Virtually any G rich motif is at least 10 standard deviations over random (data not shown). Anything more than 5 standard deviations must be considered to be experiment wide significant. It should be noted that this excess of G-rich motifs comes even though we have controlled our matched random spots to be within 5% of the GC content of the real spot they are mimicking. Across the board, our real spots have 2.5% more GC content than our random spots, well within that 5% goal. This 2.5% value can't explain the massive overrepresentation of G-rich sequences in hotspots compared to random spots. Of note, it is runs of Gs (or Cs) that we find overrepresented. Motifs with alternating G/C patterns such as GCGCGC are slightly underrepresented in real hotspots when compared to randoms.

**Table 2:** 6-mer Motif Analysis.

		LD-Defined Hotspots		Female Recombination Windows		Male Recombination Windows		deCODE Map	
	Motif	Count Excess (SD)	Clustering Score	Count Excess (SD)	Clustering	Count Excess (SD)	Clustering Score	Female K-S Score	Male K-S Score
Hotspots	GGGGGG	68.5	1.43	0.31	0.85	0.4	0.93	5.46	15.19
	GGGGGT	44.5	1.23	−0.2	0.83	0.1	0.92	4.95	14.89
	AGGGGG	41.8	1.20	−0.12	0.83	0.1	0.92	4.75	14.81
	GGGGGA	35.4	1.19	−0.11	0.85	0.3	0.92	4.42	14.48
	TGGGGG	31.2	1.18	−0.14	0.84	0.2	0.92	4.84	14.8
Windows in Females	TGGACG	1	1.03	4.4	0.85	4	0.93	5.95	14.6
	GACGTC	1.7	1.04	3.9	0.87	−1.2	0.89	5.62	14.24
	GAGCTG	−0.3	1.00	2.9	0.85	0.1	0.92	4.19	14.46
	GATCGG	0.17	1.05	2.7	0.87	0.84	0.97	4.38	14.14
	GAGCGC	0.3	1.16	2.4	0.84	1.8	0.94	6.38	15.52
Windows in Males	GGGACG	5.5	1.17	−2.5	0.86	5.5	0.92	7.12	15.81
	GGACGT	1.9	1.11	0.24	0.87	5.4	0.92	5.46	14.32
	TGGACG	1	1.03	4.4	0.85	4	0.93	5.95	14.6
	GGACGG	4.1	1.13	1.7	0.85	3.9	0.91	7.7	16.39
	CGGATG	−1.7	0.98	0.08	0.86	3.8	0.92	5	15.51
Matching deCODE Female Map	TTAGCG	0.3	1.04	−2.5	0.90	0.7	0.95	3.87	14.38
	ATGCGA	1.3	0.99	0.9	0.89	−1.7	0.95	3.89	14.35
	GGATGC	−1.4	0.97	−0.1	0.87	−0.2	0.92	3.9	14.29
	TAAGCG	0.1	1.05	−0.8	0.88	−1.2	0.94	3.9	14.32
	ATCGGT	−0.1	0.95	0.01	0.89	0.2	0.96	3.91	14.42
Matching deCODE Male Map	TGACGT	−0.7	0.98	0.6	0.88	−0.2	0.92	4.05	13.44
	TGCGTT	0.2	1.01	−1.1	0.87	0.4	0.94	4.03	13.53
	ATGCGT	0.1	1.01	−0.1	0.86	0.4	0.94	4.03	13.57
	GCGTTT	1.6	1.03	−2.6	0.87	1.5	0.95	4.15	13.58
	TGAACG	0.6	1.01	−1.2	0.87	0.4	0.94	4.07	13.58

This table is comprised of the data for 25 6-mer motifs. The rows are comprised of the motifs, each set of five chosen because they were the best identified in one of the available data sets. The columns cover each of the data sets, and detail the performance of the motif as gauged in each data set. Count Excess is the excess of motif count in real recombination windows when compared to random windows and is reported as the number of standard deviations above the mean, Clustering Score is the clustering statistic. K-S Score is the Kolmogorov-Smirnoff test statistic for a comparison of the motif distribution to the global recombination map. The score is summed over 39 chromosome arms.

These same G-rich motifs are also clustered towards the center of LD-defined Hotspots. There is a 43% (1.43 clustering score) excess of GGGGGG in the center of LD-defined hotspots over the edges. All 5 highlighted motifs show at least 18% excess in the centers of LD-defined hotspots. If there is a motif associated with the initiation of recombination this analysis would strongly argue that it is extremely G-rich.

**Figure 1:**
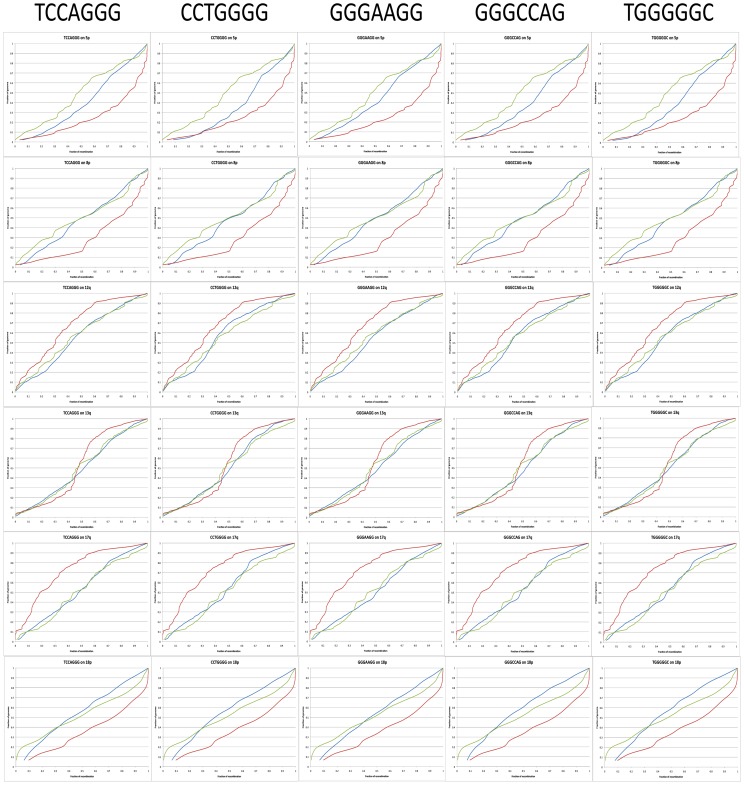
Multiple 7-mer motifs compared to deCODE recombination maps. Here, a selection of chromosome arms is offered. The motifs are labeled at the top of the figure. The rows represent each of six chromosome arms, 5p, 8p, 12q, 13q, 17q, and 18p from top to bottom. The collection demonstrates the broader concept that G-rich motifs are found nearly uniformly throughout the genome, matching well to the deCODE female map, but poorly to the deCODE male map. The y-axis displays the percentage of the genome. In each graph, the motif in question is in blue, with the line generated by treating each instance of the motif as though it were a recombination event and dividing the total to each point on the chromosome arm by the total for the entire arm. The female map is in green, while the male map is in red. The x-axis displays the percentage of recombination, the number of recombination events to that point on the arm divided by the total number on the arm.

The remaining analysis dampens this enthusiasm. Those same G-rich motifs are not enriched at all in regions of the genome observed to experience a recombination event during female meiosis. When those motifs are seen in regions undergoing recombination, they tend to cluster at the edges of the windows, not the center. They show a similar pattern in regions observed to experience recent recombination during male meiosis. They are not enriched, and if anything are clustered on the edges, not in the centers of windows. Looking at global patterns of recombination, we find these G-rich motifs show a reasonable fit to female global patterns, but no better than many other motifs (less than 4 should be considered an excellent fit (averaging close to .1 per chromosome arm, p>.37), less than 6 a good fit (p>.1), and more than 10 an awful fit (p = 0)). Compared to male global patterns of recombination the fit is simply horrible (p = 0) (Figure 1).

Moving to 6-mers that best fit the pattern of recent female recombination, we also find G-rich motifs (but somewhat less enriched than in LD-defined hotspots). The enrichment, however, is probably not experiment wide significant, and they are not clustered in the center of windows. Their fit to LD-defined hotspots is poor, their fit to global patterns of female recombination is not especially good (p values between .1 and .25), and the fit to the male pattern is horrible (p = 0).

6-mers enriched in windows of male recombination are also somewhat G-rich, and the top few are probably experiment wide significant. However, these motifs are not enriched in the center of recombination windows, are not especially enriched in hotspots (certainly no more than most G-rich motifs), do not fit global patterns of female recombination particularly well (p between .03 and .08), and fit global patterns of male recombination miserably (p = 0).

Searching for the best motifs for the global pattern of female recombination leads to 6-mers that fit that pattern exceptionally well (p between .4 and .5). However, these motifs are not remarkable in any way in any other data set. Searching for the best motif for the global pattern of male recombination does not ever result in a good fit to this data set. No single motif fits the global pattern of male recombination at all. 6-mers are too ubiquitous to fit the non-uniform distribution of male recombination events.

**Figure 2:**
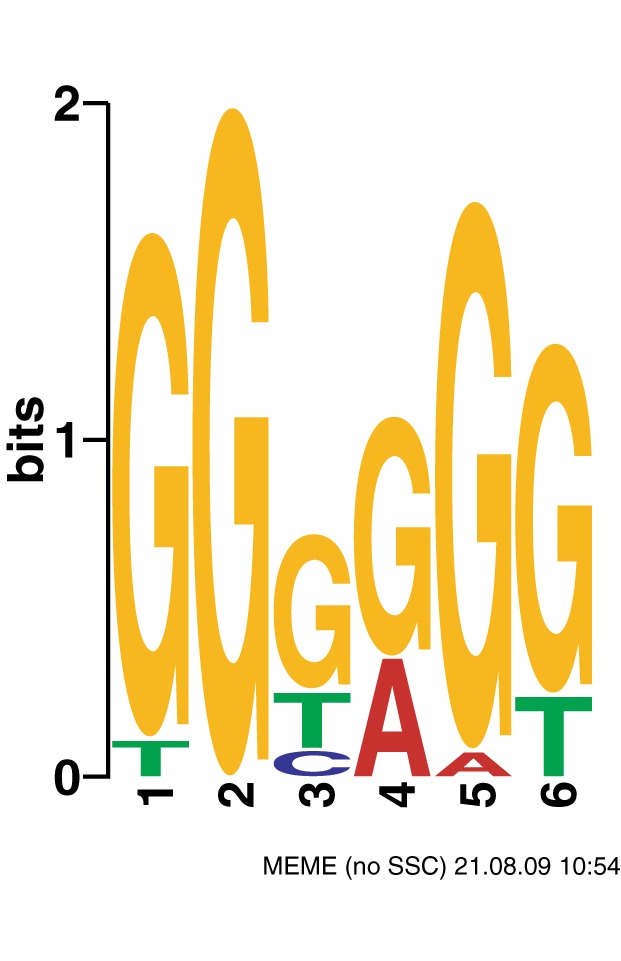
The ideal 6-mer identified in hotspots. Here we show the ideal 6-mer, identified as a composite of the top 100 overrepresented motifs in LD-identified hotspots.

In addition to testing every real 6-mer, the MEME suite [16] was utilized to generate an ideal 6-mer based on the top 100 6-mers in each data set. This ideal 6-mer (Figure 2, the 6-mer based on overrepresentation in hotspots) consists of weights for each base at each position. This ideal 6-mer has very similar characteristics to its underlying set of real motifs. It is overrepresented in hotspots. It is not overrepresented in male or female mapping windows and isn't a great fit to either deCODE map. This isn't surprising, as the ideal motif is very G-heavy, and it behaves much like the GGGGGG real motif does.

**Figure 3:**
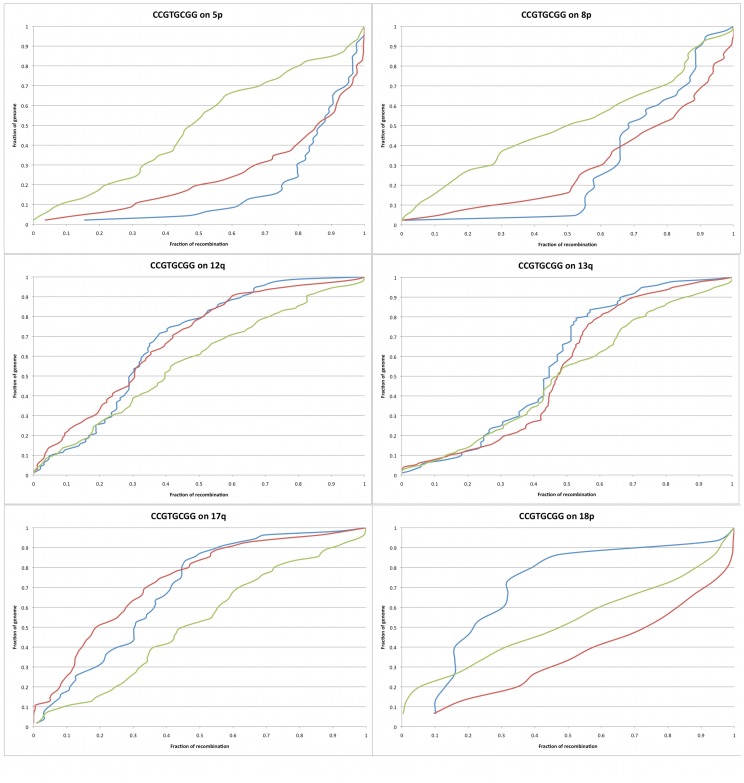
The best fitting motif on male 17q(CCGTGCGG) shown on multiple chromosome arms. Here we demonstrate that while a motif might match well to the deCODE male map on some chromosomes, on others it is terribly matched. The y-axis displays the percentage of the genome. The motif in question is in blue, with the line generated by treating each instance of the motif as though it were a recombination event and dividing the total to each point on the chromosome arm by the total for the entire arm. The female map is in green, while the male map is in red. The x-axis displays the percentage of recombination, the number of recombination events to that point on the arm divided by the total number on the arm.

Since no single 6-mer motifs fit the global patterns of male recombination well, longer motifs were examined. In an attempt to slightly reduce the data complexity, we first searched for motifs following the pattern of male recombination on 17q. 17q was chosen because it has the most telomeric pattern of recombination of all the chromosome arms, meaning that it has a higher percentage of recombination in its most telomeric 10% than any other arm. The single 8-mer, CCGTGCGG, matches best to the chromosome arm in question, and matches some other arms well, but is not a good match to all chromosome arms, and its overall KS score is above 10 (p = 0) (Figure 3). When all pairs of the same motif repeated twice are tested, GGGTGGGT spaced 20 bp apart and GCCGTG spaced 1100bp apart match well to 17q, but they too fail to repeat this matching on all chromosome arms.

Abandoning the restriction of examining 17q first, and instead searching all pairs of motifs simultaneously across all chromosomes, we find the 7-mer CCACGGG paired within 450 bases of GAGAAAC, fits the global male pattern poorly, but slightly less poorly than any single mer. Its total KS score is 8.3 (p = .008), and it fits 7 out of 39 chromosome arms well (p>.1). It appears unlikely that any motif matches the global pattern of male recombination in the genome. TGGGGAA between 100 and 200 bases from CACGCAG has a total KS score of 9.7 (p = .002), and fits 6 out of 39 chromosome arms well (p>.1). Many pairs at many combinations at various distances apart fit the data similarly well, but none could be considered to actually fit the data well.

The previously identified degenerate 13-mer follows similar patterns. The 13-mer was identified based on an original 7-mer that was overabundant in LD-defined recombination hotspots. This, however, does not make it unique. 6200 of the possible 16,384 7-mers are enriched in LD-defined hotspots. When we identify the five best 7-mers, based on their overrepresentation in hotspots, the patterns seen for them and the 13-mer are quite similar (Table 3). In all cases, between 50 and 70% of LD defined hotspots contain the motif. Each motif is found 7–12% more often in real hotspots than matched random spots. Each has between .91 and 1.75 motifs per hotspot and each finds the motif 1.2–1.5x overrepresented in hotspots when compared to random spots. Looking at the overall correlation between global recombination rates, the top five 7-mers each correlate far better with the deCODE recombination rates in each gender than does the 13-mer, with the 13-mer correlating −.006 in males and .031 in females (Table 4). The five best 7-mer motifs we have identified range from .254 to .331 in males and .197 to .326 in females. Nevertheless, it should be noted that the overall fit for all of them is atrocious in males. This data makes fairly clear that the degenerate 13-mer motif is no more convincing as an initiator of recombination than several other disparate motifs found throughout the genome.

**Table 3:** Comparisons Between Motifs in LD-Defined Hotspots.

13-mer	% of spots containing motif	Average number of motifs per spot
LD Hotspot	50	0.91
Random Spot	37.6	0.60
**GGGCCAG**		
LD Hotspot	52.5	1.19
Random Spot	45.5	0.90
**GGGAAGG**		
LD Hotspot	68.5	1.75
Random Spot	60.1	1.36
**TCCAGGG**		
LD Hotspot	56.7	1.18
Random Spot	49.9	0.93
**TGGGGGC**		
LD Hotspot	50.6	1.10
Random Spot	43.6	0.81
**CCTGGGG**		
LD Hotspot	58.3	1.61
Random Spot	49.9	1.19

This table details the presence of 6 motifs, the previously identified 13-mer and top five 7-mers, in real and randomized LD-identified hotspots. The percentages of hotspots containing a motif as well as the average number of motifs per hotspot are reported.

**Table 4:** Correlations between recombination rates and motif locations.

	Male Recombination Rate	Female Recombination Rate
**13-mer**	−.006	.031
**TGGGGGC**	.285	.197
**GGGAAGG**	.304	.326
**GGGCCAG**	.254	.222
**CCTGGGG**	.289	.209
**TCCAGGG**	.331	.289

This table presents the correlations between motif locations and the deCODE identified recombination rate in males and females for the previously identified 13-mer as well as the top five 7-mers.

## Discussion

The presence of the Chi site in E. coli has always been the basis for speculation that a motif might act in a similar fashion in humans. Although a single motif has been proposed to be recombinogenic in humans, it is anything but the definitive answer. As a result, we attempted to identify one or more motifs that were better potential options. The resultant set of analyses was enlightening, albeit in a negative way. While we were able to identify motifs that nominally match our criteria for potentially active motifs, none of them were particularly good fits. More specifically, local analyses have generated positive results in specific data sets, but global analyses have been at best non-specific in females and decidedly negative in males. Literally 1000 s of motifs roughly match the global patterns of female recombination, and virtually any G-rich motif is enriched in LD-defined hotspots.

It should be noted that human demographic history may be confounding some of this analysis [3]. As previously shown, LD-defined hotspots are enriched in high frequency SNP variation. As a result windows containing recombination events tend to be enriched for LD-defined hotspots on their edges, as these regions contain more informative markers [3], [10]. Thus, the less than 1 clustering score we observe for motifs enriched in regions of local recombination, may reflect those motifs excess presence in LD-defined hotspots, and hence enriched at the edges of recombination windows.

Importantly, however, the properties of all identified motifs are very similar to those of the identified 13-mer. That is, all of the motifs have some factors that could lead one to believe they are enriched in regions experiencing recombination, but none of them are in any way definitive. As a result, it does not appear that a motif mimicking the function of the Chi site has truly been identified as of yet. Additionally, due to the scope of our analyses, it seems unlikely that one will be identified using a computational approach similar to what we have attempted.

In the likely case that PRDM9 plays a role in generating recombination events, it is quite possible that the rapid evolution of the protein has muddled the historical set of motifs that it has used. This would explain the inability of a statistical approach to succeed. It would also raise an interesting question of whether the currently active motif locations are in new positions, relatively to previously active motifs, or whether the active motifs mutate at a high rate such that recombination occurs in the same locations over a long span. Current evidence certainly points toward the former, which would make the identification of recombination hotspots through patterns of linkage disequilibrium even less likely.

Perhaps the most intriguing result of this entire analysis is also the most impressionistic. The canonical Chi site itself is an 8-mer, and 5 of the 8 bases are G. The most clearly statistically significant result found here is that all G-rich short sequences are enriched in center of LD-defined hotspots, and the vast majority of them roughly fit the global pattern of female meiosis. One intriguing possibility is that if there is a “motif” associated with recombination in humans it is any and all G-rich short sequences, and that the specificity for G-richness is evolutionarily conserved.
